# Urinary Levoglucosan as a Biomarker of Wood Smoke Exposure: Observations in a Mouse Model and in Children

**DOI:** 10.1289/ehp.11378

**Published:** 2008-08-15

**Authors:** Christopher T. Migliaccio, Megan A. Bergauff, Christopher P. Palmer, Forrest Jessop, Curtis W. Noonan, Tony J. Ward

**Affiliations:** 1 Center for Environmental Health Sciences; 2 Department of Chemistry, University of Montana, Missoula, Montana, USA

**Keywords:** biomass, children, instillation, levoglucosan, mouse, urine, wood smoke

## Abstract

**Background:**

Biomass smoke is an important source of particulate matter (PM), and much remains to be discovered with respect to the human health effects associated with this specific PM source. Exposure to biomass smoke can occur in one of two main categories: short-term exposures consist of periodic, seasonal exposures typified by communities near forest fires or intentional agricultural burning, and long-term exposures are chronic and typified by the use of biomass materials for cooking or heating. Levoglucosan (LG), a sugar anhydride released by combustion of cellulose-containing materials, is an attractive candidate as a biomarker of wood smoke exposure.

**Objectives:**

In the present study, Balb/c mice and children were assessed for LG in urine to determine its feasibility as a biomarker.

**Methods:**

We performed urinary detection of LG by gas chromatography/mass spectrometry after intranasal instillations of LG or concentrated PM (mice) or biomass exposure (mice or humans).

**Results:**

After instillation, we recovered most of the LG within the first 4 hr. Experiments using glucose instillation proved the specificity of our system, and instillation of concentrated PM from wood smoke, ambient air, and diesel exhaust supported a connection between wood smoke and LG. In addition, LG was detected in the urine of mice exposed to wood smoke. Finally, a pilot human study proved our ability to detect LG in urine of children.

**Conclusions:**

These results demonstrate that LG in the lungs is detectable in the urine of both mice and humans and that it is a good candidate as a biomarker of exposure to biomass smoke.

The respiratory and cardiovascular health effects associated with fine particulate matter (< 2.5 μm in aerodymanic diameter; PM_2.5_) exposure have been demonstrated in large urban cohort studies (Dockery et al. 1993; Pope et al. 2002), in airsheds where there are a multitude of sources. One important source of PM_2.5_ in both developed and developing country settings is biomass combustion. People are exposed to biomass smoke in a variety of ways, including smoke from residential heating, cookstoves, campfires, forest fires, and land management (prescribed fires, controlled burns, etc.). These exposure scenarios can vary greatly with respect to intensity and duration. For example, exposures to wildland fires or agricultural burns can affect ambient air quality in nearby communities for sustained periods depending upon meteorologic conditions (Ward et al. 2005). Wood smoke from residential heating can result in moderate but sustained exposures to indoor PM (Ward et al. 2007). Such indoor exposures can be exceeded by an order of magnitude in developing countries where biomass materials are commonly used for cooking (Naeher et al. 2007; Smith et al. 2000).

Investigations of exposure to PM_2.5_ that is specifically derived from biomass combustion have been limited. Outcomes observed in intervention studies of biomass smoke health effects in developing countries include acute lower respiratory infection and chronic obstructive pulmonary disease (Smith et al. 2004). Such studies have relied on the use of novel personal and area monitoring devices suitable for high-level PM exposures (Chowdhury et al. 2007). However, data are limited with respect to additional putative health effects associated with moderate and/or intermittent exposures to biomass smoke. Exposure assessment for population-based studies in these settings can be imprecise when they depend on ambient or fixed-site monitoring, or cost-prohibitive and logistically cumbersome when using personal monitoring. The animal and human data presented in this article support the use of a novel biomarker of exposure to wood smoke that could be useful in future epidemiologic studies assessing human health effects of biomass smoke-derived PM_2.5_ in these varied exposure scenarios.

The ideal biomarker would be a dose-dependent indicator of exposure, in addition to a predictor of physiologic effects, and obtained through noninvasive techniques. However, a more realistic goal would be the combination of a marker of exposure and a separate marker of effect. Although others have pursued the use of compounds such as methoxyphenols for biomarkers of wood smoke exposure (Clark et al. 2007; Dills et al. 2006), current data in our laboratory have produced several other potential candidates, including levoglucosan (LG).

Levoglucosan (1,6-anhydro-β-d-glucopyranose) is a pyrolysis product of cellulose and is one of the major organic components of ambient PM emitted from biomass combustion. It is frequently used as a tracer for biomass burning because it is produced at relatively high levels and is stable in the atmosphere (Fraser and Lakshmann 2000; Simoneit et al. 1999). During the Montana forest fire season of 2003, LG concentrations in the range of 900–6,000 ng/m^3^ were observed from quartz filter samples collected in the Missoula valley (Ward et al. 2006a). Although these values are generally higher than those detected in other U.S. urban areas, including Seattle, Washington [13–760 ng/m^3^ (Simpson et al. 2005)], Spokane, Washington [2–327 ng/m^3^ (Jimenez et al. 2006)], and Fresno/Bakersfield, California [23–7,590 ng/m^3^ (Schauer and Cass 2000)], they were comparable to LG concentrations measured during the dry season at Rondonia, Brazil [446–4,106 ng/m^3^ (Zdrahal et al. 2002)] when intensive biomass burning was occurring. However, far higher concentrations have been measured during severe episodes of biomass smoke pollution in Southeast Asia [1,400–40,240 ng/m^3^ (Radzi bin Abas et al. 2004)]. LG constituted 2.8–3.8% of PM_2.5_ mass from open burning of foliar fuels (Hays et al. 2002) and 5.7% of PM_2.5_ mass emissions from prescribed burns of forests in Georgia (Lee et al. 2005). Previously reported values of the ratio of LG to PM in fireplace emissions ranged between 0.8% and 26% (Fine et al. 2001, 2002, 2004). In Libby, Montana, where the airshed was heavily affected by the use of non–EPA wood stoves not certified by the U.S. Environmental Protection Agency (EPA) during the winter of 2003/2004, LG comprised 10% ± 2% of the wintertime PM_2.5_ (Ward et al. 2006b), averaging 2.8 μg/m^3^ throughout the winter months.

Because of its abundance in biomass smoke and a positive correlation with levels of PM_2.5_ mass, LG is considered a good candidate for assessment as a biomarker of exposure to wood smoke. LG is specific for wood combustion, and exposures to wood smoke constitute considerable health concerns both in the United States and around the world. The ability to evaluate an individual exposure noninvasively would be a powerful tool for both observational epidemiologic studies and intervention studies. In this article, we present a method of LG detection in urine that we used in initial experiments to assess the detection of LG from multiple instillations and exposures in animals that included pure LG, concentrated wood smoke particulates, and wood smoke inhalation. The present study was designed to achieve three primary steps in the development of LG as a biomarker: *a* ) detect LG in mouse urine after respiratory exposure; *b*) determine specificity to wood smoke; and *c*) detect LG in human urine. The findings presented here have effectively achieved these aims and set the basis for future, more rigid human studies to determine the usefulness of LG as a biomarker of exposure to wood smoke.

## Material and Methods

### Mouse model

#### Mice

We used Balb/c mice (Jackson Laboratory, Bar Harbor, ME) for all *in vivo* studies. The Balb/c strain is used for a wide variety of studies and has been well characterized in multiple models. Animals were housed in microisolators on a 12/12-hr light/dark cycle. The mice were given food and deionized water *ad libitum*. Mice were treated properly to minimize discomfort and suffering, and all animal procedures were approved by the University of Montana Institutional Animal Care and Use Committee.

#### Instillations

For intranasal instillations, animals were anesthetized with 0.1 cc ketamine [1:4 in sterile phosphate-buffered saline (PBS)] by intraperitoneal injection. Mice were then instilled with 25 μL of the specified treatment. Treatments included 1,6-anhydro-β-d-glucopyranose, glucose, or concentrated particulates, in PBS. The treatment regimen consisted of a single instillation followed by urine collection within 24 hr. Initially, three amounts (250 μg, 25 μg, and 5 μg) of LG were instilled in mice. Urine samples were collected at 2-, 4-, 6-, and 8-hr time points and pooled for analysis. A second set of instillations used 250 μg LG for a time course study. We collected samples at 0, 1, 2, 4, 6, 8, 10, and 24 hr postinstillation. For the third set of instillations, which was conducted to assess the specificity of LG compared with glucose, we instilled 250 μg and pooled the 2-, 4-, and 6-hr collections for analysis.

#### Directly instilled PM

Mice were directly instilled with several kinds of PM: *a*) PM_2.5_ harvested from the ambient air of Missoula, Montana; *b* ) wood smoke PM_2.5_ harvested from a non–EPA-certified woodstove; and *c*) diesel exhaust PM (DEP). Particles were weighed, resuspended in sterile PBS, and sonicated in a water bath for 1 min immediately before instillations. Mice were instilled with 125 μg particles, and urine was collected at 2, 4, and 6 hr postinstillation.

#### Ambient air PM

We used a versatile aerosol concentration enrichment system (VACES) (Kim et al. 2001) particle concentrator to harvest PM_2.5_ from the ambient air in Missoula. Air samples were collected by the concentrator on the roof of a three-story building at the University of Montana. The concentrator had three parallel sampling lines (concentrators) that simultaneously collected fine PM, each at a set flow rate of 110 L/min. We concentrated the fine fraction (PM_2.5_) by drawing air samples through two parallel lines, using 2.5 μm cut-point preimpactors to remove larger particles. These particles were then drawn through a saturation-condensation system that grows the particles to 2–3 μm droplets before being concentrated by virtual impaction. Particles and the water-soluble fraction were then collected in glass impinger Biosamplers (SKC, Inc., Eighty Four, PA), and then lyophilized to harvest the ambient PM_2.5_.

#### Wood smoke

We used the VACES particle concentrator to collect smoke PM_2.5_ emitted from a non–EPA-certified wood-stove. In a controlled simulation, a non–EPA-certified woodstove (Englander; England’s Stove Works, Inc., Monroe, VA) was loaded with a mixture of locally obtained softwoods (Douglas fir, larch, and Ponderosa pine), with the smoke pumped into a modified inhalation chamber to allow the smoke to cool and age (residence time was no more than 2 min). The combustion conditions during the burns ranged from flaming to smoldering. The VACES was then used to harvest smoke PM_2.5_ into a deionized water dropout (Biosampler; SKC, Inc.). At the conclusion of the smoke PM harvesting trial, two fractions were collected: a water-soluble fraction and a water-insoluble black, tar-like material that coated the inside of the impinger. Because we were unable to get the water-insoluble fraction in a suitable solution with which to instill mice, we used only the water-soluble fraction of the harvested PM_2.5_ in this study. However, using gas chromatography/mass spectrometry (GC-MS), we determined that only the water-soluble fraction contained detectable levels of LG (data not shown).

#### DEP

For this study we used standard reference material (SRM) for diesel PM [SRM 1650; National Institute of Standards and Technology (NIST), Gaithersburg, MD].

#### Smoke exposure

Wood smoke emitted from an older-model, non–EPA-certified woodstove was directed by aluminum flex tubing into a modified inhalation chamber. PM_2.5_ concentrations inside the chamber were regulated using several in-line valves, with continuous PM_2.5_ measurement conducted using a TSI DustTrak (TSI, Inc., Minneapolis, MN). We used the same type of wood and burn conditions described above for wood smoke. Fires were started with 4 g paper and 20 g kindling, and maintained by adding pre weighed wood batches (50.00–54.99 g) approximately every 5 min. Briefly, mice were placed in individual slots in an animal housing unit within the exposure chamber, which is composed of perforated metal to allow diffusion of wood smoke through the compartments. Mice were exposed in two separate groups for 2 hr each at a target concentration of 3–4 mg/m^3^. During the combustion process, temperature and carbon monoxide readings were constantly observed. Procedures are approved in Animal Use Protocol 050-06 at the University of Montana.

#### Urine sample collection

Urine samples were collected in sterile 1.5-mL Eppendorf tubes at designated time points. For “pre” samples, urine was collected just before intra-peritoneal injection. Briefly, one researcher handled the animals by grasping them from behind and positioning them such that a second researcher collected the fluid in prelabeled tubes. Samples were stored at −20°C until analyzed.

### Human pilot study methods

#### Human subjects

We used a convenience sample of 14 grade-school children in Libby, Montana, to evaluate the presence of LG in urine. All were non-Hispanic white children with a mean age of 8.5 years (range, 7–10 years of age). The residences of these subjects were located within the Libby airshed, an area with moderately elevated levels of ambient wood smoke from wintertime domestic woodstove use. We collected information from parents on the type of home heating and whether tobacco smokers were in the household. Spot urine samples were collected at the school between 0830 and 1400 hours, aliquoted, and stored at −80°C. Eight samples were collected in the morning (i.e., before noon) and six samples were collected in the afternoon. All human sample collection procedures, including documentation of parental permission and child assent, were approved by the University of Montana Institutional Review Board.

#### Indoor and ambient air monitoring

Although this study was not an experimentally designed exposure study, we include air monitoring data in order to describe the environmental context of these subjects’ potentials for exposure to wood-smoke–derived PM. We conducted indoor air monitoring at the children’s school and ambient air monitoring at a central site located < 0.25 mile from the school. A Sioutas impactor PM sampler with Leland Legacy pump (SKC, Inc.) was fitted with Teflon filters to measure the gravimetric mass of five size fractions of the indoor PM: > 2.5 μm, 1.0–2.5 μm, 0.5–1.0 μm, 0.25–0.5 μm, and < 0.25 μm. A collocated PM_2.5_ cyclone (BGI, Inc., Waltham, MA) was fitted with a 47-mm prefired quartz filter for subsequent analysis of specific chemical markers of wood smoke, including LG. We used a GC-MS method developed in our laboratory (Bergauff et al. 2008) to extract analytes from the filters. With this method, recovery of LG averaged 96%, with a typical relative standard deviation of 10%. Ambient PM_2.5_ data were collected from the Montana Department of Environmental Quality’s PM_2.5_ compliance site for the town of Libby, located approximately 0.5 mile from the school.

### Urine sample analysis

#### Sample preparation

For analysis of the urine samples, we used a GC-MS method developed in our laboratory, based on a previously published method (Tetsuo et al. 1999). Briefly, a 50-μL aliquot of each mouse urine sample was placed in an Eppendorf tube, approximately 30 U urease (Sigma, St. Louis, MO) was added, and then incubated at 37°C for 30 min. Samples were then spiked with D7-levoglucosan as a standard, and 500 μL ethanol was added. Samples were centrifuged and the supernatant was transferred to another Eppendorf tube to remove excess protein. Samples were then dried overnight in a vacuum manifold. Each sample was then mixed with 50 μL distilled water and frozen. Samples were then placed on a freeze dryer for ≥ 3 hr to remove any remaining liquid. The dry samples were then derivatized with 75 μL *N*,*O*-bis(trimethylsilyl) trifluoroacetamide, 10 μL trimethylchlorosilane, and 10 μL trimethylsilylimidazole (Sigma) and heated for 1 hr in an oil bath at 70°C. Samples were diluted to 250 μL with ethyl acetate containing 3.6 mM triethylamine (Fisher Scientific, Hampton, NH) and centrifuged to remove any remaining solids. Approximately 100 μL of each sample was then transferred to a GC vial for analysis. Human urine samples were analyzed with the same procedure except that 100 μL of sample was used and treated with 900 μL ethanol. The final dilution for each human urine sample was 0.5 mL; the deuterated standard spikes were adjusted accordingly. We also analyzed human urine samples by enzyme-linked immunosorbent assay (ELISA) for both creatinine (Cayman Chemical Co., Ann Arbor, MI) and cotinine (Calbiotech, Spring Valley, CA). LG and cotinine results for human samples were adjusted for urinary creatinine.

We prepared calibration standards containing 10, 25, 50, 80, or 125 ppm LG with a fixed concentration of 80 ppm d-levoglucosan (Cambridge Isotope Laboratories, Andover, MA) as an internal standard, spiked into a blank urine sample. The D7-levoglucosan had a mass of 169 Da before derivatization, compared with 162 Da for LG. Blank urine was collected from mice that had not been exposed to wood smoke or other potential sources of LG. We analyzed potential blank samples using the GC-MS method to verify the absence of LG. Blank samples from several mice were combined, and this pool was used as the matrix for the calibration curves. The standards were derivatized and analyzed on the GC-MS according to the procedure described above. We prepared a calibration curve by plotting the ratio of the two peak areas versus the concentration of the tracer, and the *R*^2^ value was 0.9634. The concentration of analytes in the samples was determined by measuring the ratio of the peak area for the analyte to that of the corresponding deuterated standard, and reading the concentration from the appropriate calibration curve. Blanks of distilled water were analyzed daily with the samples to monitor for contamination during analysis, and none tested positive for LG (*n* = 12). We chose distilled water as the matrix for blank because of the limited volume of blank mouse urine available. We also prepared spikes in water with the samples. Average recovery of LG was 107 ± 9.5% (*n* = 15). Detection limits for the method were defined as the concentration of analyte that gives an instrument response that is three times the standard deviation of the instrumental baseline signal. The detection limit for LG in the final ethyl acetate extract was 0.92 μg/mL (1.8 ng injected, 37 pg on-column), which equates to a detection limit of 0.23 μg in 50 μL of urine sample with the dilutions used in our method.

### GC-MS analysis conditions

We performed the analysis on an Agilent 6890N gas chromatograph with an Agilent 5973 mass spectrometer (Agilent Technologies, Santa Clara, CA). We used an HP-5MS column [(5% phenyl)-methylpolysiloxane] with dimensions of 0.25 mm i.d., × 30 m length × 0.25 μm film thickness. A volume of 2 μL was injected for each analysis into a split/splitless FocusLiner for HP (Supelco, Bellefonte, PA), single taper packed with quartz wool liner. Split injection was used to analyze for LG, with a split ratio of 50:1. Helium was used as the carrier gas at an initial flow rate of 1 mL/min through the column. The inlet temperature was set at 250°C and the auxiliary transfer line temperature was set at 280°C. The temperature program was started at 40°C for 1.5 min, ramped at 30°C/min to 175°C, 20°C/min to 220°C, held for 2 min at 220°C, and then ramped at 50°C/min to a final temperature of 300°C, which was held for 1.5 min for a total run time of 13.95 min. We operated the mass spectrometer with a solvent delay of 4.00 min and scanned the mass range from 40 to 450 Da. For all compounds, highly selective quantitation was performed using the signal for representative ions extracted from the total ion chromatogram. LG was analyzed using an *m*/*z* of 217, whereas *m*/*z* 220 was used for D7-LG.

### Statistical methods

All data were analyzed using SAS, version 9.1 (SAS Institute, Inc., Cary, NC). Urine LG and urine creatinine data were log transformed to approximate normality. We assigned mouse urine samples with undetectable levels of LG a value of half the detection limit, or 0.12 μg for a 50-μL urine sample. Data were analyzed by analysis of variance and *t*-test as appropriate. Urine LG and urine cotinine were compared by Pearson correlation. The descriptive urine LG data presented in the text, tables, and figures were untransformed.

## Results

### Mouse model results

#### Detection of instilled LG in urine

**Figure 1:**
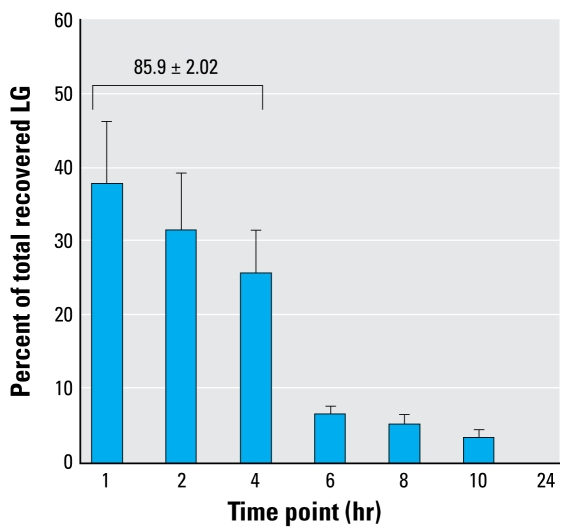
LG recovery in mouse urine over time in mice instilled intranasally with 250 μg LG in PBS. The total amount of recovered LG was determined for each animal, and the percent of total recovered LG was calculated for each mouse at the specified time point. Values are mean ± SE of 11 animals. The value for percent recovered in the first 4 hr was calculated by summing the first three values (1, 2, 4 hr) for each mouse and then finding the mean ± SE of those 11 values.

After intranasal instillation, the higher doses (250 μg and 25 μg) averaged about 40% recovery of the total LG instilled. The lower dose (5 μg) appeared to be below the level of detection and did not result in consistent values. For the time course assessment, we detected no LG in the “pre” and “24 hr post” samples (Figure 1), but LG was detected at all other time points (1–10 hr). In this study, we recovered an average of > 50% of the instilled LG (data not shown). However, most of the recovered LG was recouped by 4 hr (> 85%). These results indicate that LG is detectable in urine after introduction to the lungs and that a significant amount is recovered within the first 4 hr.

#### LG detection specificity

**Figure 2:**
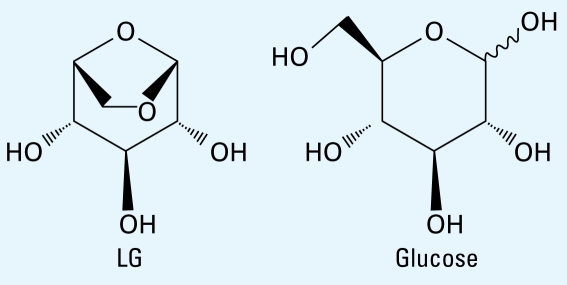
Comparative structures of LG and glucose.

**Figure 3:**
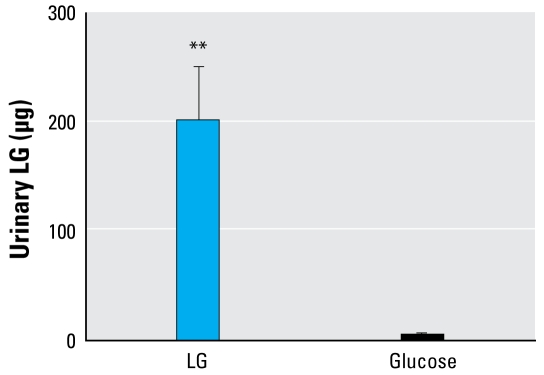
LG detection specificity in mice instilled intranasally with 250 μg LG or glucose in PBS. Urine samples were collected at 0, 2, 4, and 6 hr and the three postinstillation samples were pooled for analysis by GC-MS. No LG was detected in the 0 time point or preinstillation samples (data not shown). Values are mean ± SE of five animals. **p < 0.01 by Student’s *t*-test.

Because of the high structural homology between LG and glucose (Figure 2) and a potential for glucose to be metabolized to LG, we performed a direct comparison between the two sugars. We detected no LG in “pre” instillation samples from either group. One mouse from the glucose-instilled group had detectable levels of LG, but it was significantly lower than that detected in the LG-instilled mice (Figure 3). As expected, all four LG-treated mice had high urinary LG levels. These data confirm that glucose is not metabolized into LG and does not interfere with the analysis.

#### Concentrated particulate instillation

**Figure 4:**
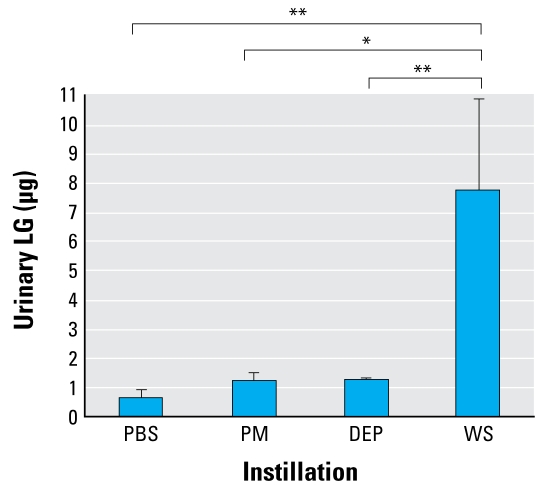
Urinary LG detection in mice instilled intranasally with 125 μg of particles from Missoula ambient air (PM), DEP, or wood smoke (WS). Urine samples were collected at 0, 2, 4, and 6 hr, and the three postinstillation samples were pooled for analysis by GC-MS. No LG was detected in the 0 time point, or preinstillation samples (data not shown). Values are mean ± SE of 5–10 mice. *p < 0.05; and **p < 0.01 by Student’s *t*-test.

The data in Figure 4 show that the levels of LG detection in the PM- and DEP-treated mice were slightly above that in the PBS controls, whereas the wood-smoke–treated mice had significantly higher levels of LG detected in the urine. The average amount of LG recovered (8.01 μg) was about 24% of that instilled, assuming that each mouse was instilled with 33.25 μg LG (26.6% of 125 μg). These data indicate that LG at sufficient levels in wood smoke PM can be detected in urine after intranasal instillation and is a marker specific to wood smoke compared with other sources of PM.

#### LG detection after wood smoke inhalation

**Figure 5:**
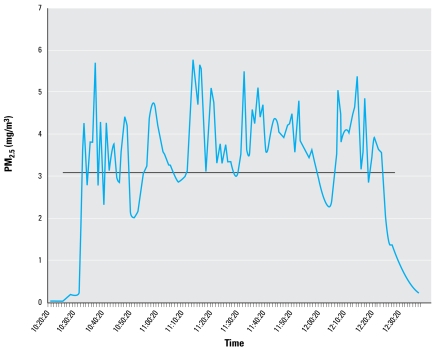
Real-time PM_2.5_ monitoring during a wood smoke exposure shown in 60-sec increments over a 2-hr exposure. See “Materials and Methods” for details. The black line indicates the duration and overall PM_2.5_ average of 3.142 mg/m^3^.

**Figure 6:**
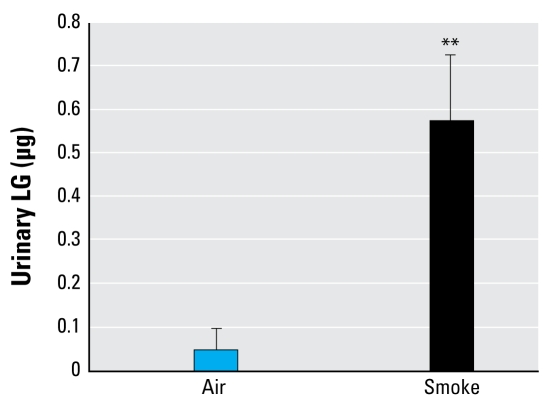
Urinary LG detection in mice exposed to to air only (*n* = 14) or to 2–4 mg/m^3^ of wood smoke PM_2.5_ (*n* = 13) for 2 hr. The values are averages of 13 (smoke) or 14 (air) ± SE. No LG was detected preexposure (data not shown). **p < 0.01 by Student’s *t*-test.

For the wood smoke inhalation experiments, one exposure averaged 3.14 mg/m^3^, and the second exposure averaged 3.75 mg/m^3^. Figure 5 presents a representative graph of the time course of PM_2.5_ mass concentration in 1-min increments. The combination of the two separate exposures is summarized in Table 1. Only 1 of 14 air-exposed controls was positive for LG; 10 of 13 (76.9%) samples collected from smoke-exposed mice contained detectable levels of LG (Figure 6). In calculating particle deposition, we assumed a minute ventilation (mv) of 42.6 cc/min and a minimum 20% deposition (*d*) (derived empirically from PM_2.5_ deposition models) based on previously published studies (Hsieh and Oberdorster 1999; Kleeman et al. 1999). Taking the average exposure (*ae*) of 3.14 mg/m^3^ for 2 hr (*t*), we calculated that approximately 3.2 μg PM_2.5_ was deposited in the lungs of exposed mice [(*ae*) × (*mv*) × (*t*) × (*d*) = amount deposited]; based on an LG concentration of 26.6% (Table 2), each mouse was potentially exposed to 0.85 μg LG. The average LG recovered from the 13 smoke-exposed mice was 0.574 μg (Figure 6), or approximately 67% (0.574 μg/0.85 μg) of the calculated exposure. These results support the use of LG as a specific biomarker of exposure to wood smoke.

### Human pilot results

#### Ambient and indoor conditions in sampling area

On the day of the urine collection, ambient PM_2.5_ mass was 5.9 μg/m^3^, PM_2.5_ mass inside the school was 41.1 μg/m^3^, and LG in the particulates inside the school was 98.5 ng/m^3^.

#### Urinary LG in children

We detected LG in all 14 urine samples. The mean ± SD creatinine-adjusted LG concentration was 55 ± 94 ng/mg creatinine. Urinary LG concentrations by selected factors are presented in Table 3. Woodstoves were reported as the primary heating source for 9 of the 14 homes. Average urinary LG among children living in homes with woodstoves was slightly higher than among children living in homes without woodstoves, but this difference was not significant. Smoking was reported in 6 of the 14 homes, and children’s urinary LG was associated with parent-reported household smoking (*p* = 0.003). Comparisons of urinary LG by the presence of woodstove or tobacco smoking in the household did not change when limiting the analyses to the eight children whose samples were collected in the morning (data not shown). We evaluated urinary cotinine to further assess the association with exposure to environmental tobacco smoke, but urinary cotinine concentrations did not correspond with reported household smoking. Urinary cotinine was 28 ± 30 ng/mg creatinine among the six children living in homes with reported smoking and 22 ± 44 ng/mg creatinine among the eight children living in homes with no reported smoking (*p* = 0.27). We did not find strong correlation between urinary cotinine and urinary LG concentrations (*r*= 0.27, *p*= 0.36).

## Discussion

Chronic (Bates et al. 2007; Mishra et al. 2004; Ward et al., in press) or episodic exposures to biomass smoke present potential health concerns. Intermittent exposures to high levels of biomass smoke can have effects on multiple aspects of human health, including exacerbation of asthma and cardiovascular disease and alterations in either pulmonary or systemic immunity. We designed the present study to ascertain the potential for LG as a biomarker of exposure to wood smoke. We chose to target LG because it is one of the major organic components in ambient PM from biomass combustion, and it is usually found at much higher concentrations than are methoxyphenols. LG is frequently used as a tracer for biomass burning because it is produced at relatively high levels and is stable in the atmosphere (Simoneit et al. 1999). Because of the potential health concerns for wood smoke inhalation, the development of a specific biomarker would be of great importance in assessing the health effects of exposed individuals or communities. To determine the feasibility of LG as a biomarker, we needed to demonstrate that our method was specific for the sugar, that it was specific for wood smoke exposures, and that it can detect LG in urine from human subjects. All of these criteria were sufficiently met in the animal models, according to the data within this study, and the human data support the method of detection.

A major issue with a biomarker of exposure is that of metabolism. Therefore, initial studies revolved around the ability not only to detect LG in the urine after introduction in the alveolar spaces, but also to determine recovery. Although only 24% was recovered in the wood smoke PM instillations (Figure 4), a minimum level of 40% recovery was obtained in both pure LG instillation (data not shown) and wood smoke inhalation (Figure 5) studies. We saw a greater yield in the glucose comparison study (~ 80% average; Table 2). This may indicate the range of our system and may depend on the timing of sample collection. Although we collected urine samples at specific time points, the actual amounts vary between animals and time points. The other question of metabolism considers the potential of other sugars of similar structure being metabolized to LG. Although the metabolism of glucose is well understood, these experiments showed that it is not metabolized to LG, so the LG detected in the urine is from instilled LG only (Table 2). These data further illustrate the specificity of our LG detection method.

LG has been used as a molecular marker of wood smoke in atmospheric tracking studies (Fraser and Lakshmann 2000), and LG is a well-documented component of wood smoke PM (Dills et al. 2006; Fine et al. 2001, 2002; Hedberg et al. 2006). Although there is a wide range of reported levels, our concentrated wood smoke particulates in the experimental setting contained 26% LG (Table 1). Ambient air samples contained < 0.5% LG, and we detected no LG in DEP. All of these reported levels from a variety of sources correlated in the urinary detection of LG; for example, we detected no LG in the urine of mice instilled with DEP, whereas all mice instilled with wood smoke PM had detectable levels. In addition, more than three-fourths (76.9%) of the mice exposed to wood smoke in the inhalation studies had detectable levels of LG in the urine. Although the contribution of particle ingestion (i.e., from grooming) cannot be discounted, the main objective of these studies was to prove specificity, and the instillation experiments support the hypothesis that LG in the lungs can be detected in urine. We did not detect LG in the urine from three smoke-exposed mice, but this is likely resulted from an error in manual urine sampling, rather than continual sampling that would occur in a metabolic cage. Another concern is the fact that one air-only–exposed animal had detectable LG in its urine sample. However, it was only 1 of 14 air-only–exposed animals, and the level detected (0.649 μg) was still below the average level detected in PBS-instilled controls (0.788 μg) in Figure 4.

We detected LG in the urine samples from all 14 children, suggesting the potential for further investigation of this biomarker in humans under controlled experimental settings or in observational studies with rigorous exposure assessment. Urinary LG in these children could not be clearly associated with the presence of a woodstove in their homes, because we did not have direct exposure information for these children, and we were unable to determine the degree to which woodstoves were used in their respective homes in the days leading up to the sampling event. We did not have personal PM exposure information for these children, but our previous studies in this community have demonstrated the potential for high wood-smoke–derived ambient PM_2.5_ concentrations (Ward et al. 2006b) as well as high indoor PM_2.5_ concentrations in the grade school attended by the child subjects (Ward et al. 2007). Such sources of PM exposures outside the home may have diluted any effect with woodstoves that we may have otherwise been able to detect. The finding that urinary LG concentrations were slightly higher among those children that provided a sample in the afternoon rather than the morning suggests the potential influence of ambient or in-school exposures on urinary LG, but it is difficult to draw conclusions on this point with the limited number of observations. Although the kinetics of human LG exposure remains to be determined in experimental settings, these convenience samples from children suggest the potential for this biomarker to reflect exposures to wood-smoke–derived PM from a variety of sources. In settings such as Libby, where wood smoke is the predominant contributor to ambient PM, a biomarker that integrates both in-home and ambient exposures would be a useful tool for large-scale epidemiologic studies compared with more costly personal air monitoring.

Our findings for environmental tobacco smoke exposure and urinary LG were inconsistent. Parent-reported smoking in the home was strongly associated with urinary LG, but a biomarker of exposure to tobacco smoke was not associated with urinary LG. It is possible that exposure misclassification occurred when we based exposure to tobacco smoke on parent report. Also, the pharmacokinetics of cotinine are likely quite different from those of LG. Indeed, our findings suggested that most LG is excreted within a few hours, whereas the half-life for cotinine is close to 1 day (Heinrich-Ramm et al. 2002). The varied urine sample collection times could account for the discrepancy in our findings with respect to parent-reported smoking versus biochemical evaluation of children’s exposure to environmental tobacco smoke. Nevertheless, tobacco smoke is a potential source of LG (Sakuma et al. 1980) and should be taken into account when evaluating urinary LG as a biomarker of exposure to other sources of biomass smoke.

In summary, the present study presents strong evidence for the use of LG as a biomarker of wood smoke exposure. Both direct instillation and whole-body inhalation exposures appeared to have similar kinetics for detection in mouse urine. Also, the descriptive human data presented here demonstrated that LG can be detected in children’s urine samples. Further work is necessary to evaluate the relationship between LG levels and exposure to tobacco smoke, determine the time- and dose-sensitive kinetics of LG exposure and excretion, and investigate any additional factors such as diet that may affect LG levels. The next step in the process will be to further characterize this biomarker in exposed humans and to use it in experimental and population-based studies of wood smoke exposure and human health effects.

## Figures and Tables

**Table 1:** Summary of separate inhalation exposures, urine sample collection, and analysis.

Exposure	No. of mice	Urine samples collected	Positive for LG
Air only 1	6	6/6	0/6
Air only 2	10	8/10	1/8
Total	16	14/16	1/14
Smoke 1	6	6/6	4/6
Smoke 2	11	7/11	6/7
Total	17	13/17	10/13

**Table 2:** Comparative LG levels in concentrated PM from multiple sources.

Particle source	Concentration in PBS (mg/mL)	Percent LG
Ambient air	5	0.42 ± 0.006
DEP	5	0.0
Wood smoke	5	26.6 ± 1.4

a Values are mean ± SE of three separate analyses.

**Table 3:** Urinary LG in children by selected factors (mean ± SD).

Factor	No.	Urinary LG (ng/mg creatinine)	*p*-Value^a^
Sex			
Female	2	18.9 ± 23.6	0.27
Male	12	61.2 ± 100.0	
Woodstove in home			
No	5	42.9 ± 48.8	0.89
Yes	9	62.0 ± 113.4	
Time of sample collection			
Morning	8	30.6 ± 42.9	0.09
Afternoon	6	87.9 ± 133.8	
Smoking in home			
No	8	14.3 ± 11.1	0.003
Yes	6	109.6 ± 127.7	
Urinary cotinine (ng/mg creatinine)			
< 10	7	66.6 ± 130.2	0.54
≥10	7	43.8 ± 40.8	
Total	14	55.2 ± 93.5	—

a Test on log-transformed values.
